# Association between overweight, obesity and incidence of advanced dental caries in South Korean adults: A 10-year nationwide population-based observational study

**DOI:** 10.1371/journal.pone.0229572

**Published:** 2020-02-27

**Authors:** KyungJae Kim, Kyungdo Han, SungEun Yang

**Affiliations:** 1 Department of Conservative Dentistry, College of Medicine, Seoul St. Mary’s Dental Hospital, The Catholic University of Korea, Seoul, Korea; 2 Department of Biostatistics, College of Medicine, The Catholic University of Korea, Seoul, Korea; International University of Health and Welfare, School of Medicine, JAPAN

## Abstract

The objective of this study was to evaluate the association between overweight, obesity and the incidence of advanced dental caries in South Korean adults, using alternate measures. The participants included 376,077 people aged 20 years and older who had health examination at least one time between 2005 and 2008. This evaluation is based on a change of body mass index (BMI) category, for 10 years, using a nationally representative data resource available from the National Health Insurance System. Instead of using decayed, missing, and filled teeth (DMFT), the diagnostic codes which indicate dental caries, pulpal disease and visiting frequency at dental health professionals were used in this case. A multivariate adjusted Cox regression analysis was performed to examine the association between advanced dental caries and BMI. In addition to the BMI, a multivariate analysis of gender, age, lifestyle behaviors and systemic disease information was included. To this end, the hazard ratio (HR) and 95% confidence interval (CI) were calculated. Chiefly, it is noted that the overweight and obese people were more likely to develop advanced dental caries independent of the noted variables. The positive association between high BMI and incidence of advanced dental caries was more prominent in the population’s characteristic of people who were in a classification of the elderly and women. Among the health and lifecycle behaviors, smoking or not was found to be one of the factors affecting the results. The alternate method used in this study showed that being overweight and obesity had a direct association with the incidence of advanced dental caries in Korean adults.

## Introduction

Dental caries is a multifactorial disease that affects most of the world’s population and is a detrimental factor to the preservation of an individual’s oral health for a lifetime. It is the primary cause of oral pain and tooth loss as noted in adults and children [1]. Although dental caries has shown a declining trend over the past few decades, recent studies have indicated that it is on the increase once again nowadays because of many factors affecting health [2]. According to a systematic analysis for global burden of diseases in 2015, dental caries, especially permanent caries, was ranked the first in prevalence and the third in incidence among all chronic diseases in a general sense [2]. Its incidence was increased by 15% as compared to that in 2005 [2]. In various fields, efforts have been made to prevent these problems by recognizing deterioration of oral health and dental caries levels to provide awareness to public health, in an effort to develop strategies to prevent the increase in dental caries with better health outcomes for all patients. Despite attempts of WHO and many experts, the continued increased consumption of refined sugar and the excessive consumption of foods lacking healthy nutrition have resulted in weight gain and increased incidence of dental caries in some countries globally [3,4].

National, subnational, and multi-center studies have shown that obesity, as measured by body mass index (BMI), has increased in recent decades in many populations globally [5]. The incidence of being overweight or obese is linked to an increased risk for several chronic diseases, including diabetes, heart disease, and cancer [6]. People who are overweight may also experience health issues that may be linked to oral health, especially with an increased risk of developing dental caries. These studies show that lifecycle behaviors such as smoking, alcohol consumption and physical exercise are also associated with BMI, but there is a lack of analysis of the association with dental caries [7–10]. The significant factors affecting the expression of dental caries include tooth, time, bacteria, and the individual’s diet [11]. Among this, time and diet factors are shown to be directly related to overweight and obesity. It is easily anticipated that a higher incidence of dental caries will be observed in higher BMI populations, and many of these associations, especially in children, have been studied in the past. Several studies have showed that children who experience dental caries are more likely to have weight increases in their patient histories. Hayden et al. [3] have reported a positive association between obesity and dental caries in children through a meta-analysis and review of this data. Previous studies have only confirmed the link between BMI and the number of teeth or DMFT (decayed, missing, and filled teeth) index [12,13]. South Korea's obesity rate is the lowest among Organization for Economic Cooperation and Development (OECD) countries, but has been following a trend of having steadily increased over the past few decades. About 5.3 percent of the adult population are obese, and upwards of 31 percent of citizens are considered to be overweight (including the diagnosis regarding obesity) in Korea. Although previous study has reported a decrease in the prevalence rate of dental caries in South Korea, the study also has used the DMFT index to make this determination [14].

The incidence and placement of dental caries usually begins at the surface of the tooth. It affects enamel, dentin, and pulp. Its treatment and diagnosis are managed differently for each tooth depending on its severity. When caries is mild and confined to the tooth surface, the pulp is normal and restorative treatment can be used. However advanced caries can cause pulpitis or pulp necrosis, thus requiring root canal treatment. The incidence of dental caries can be classified in various ways. It is usually based on a review of clinical and radiological results. Advanced dental caries means lesion that there is a lesion that extends to or through the dentinoenamel junction without extending more than half the distance to the pulp. If caries progresses to this stage, most patients are considered to be symptomatic. The dentist will consider root canal treatment at that stage of dental decay [15–18]. Whether or not root canal treatment is implemented in clinical practice is important for both the patient and the clinician. The vitality of the pulp, the need for further crown restoration, and the long-term prognosis are all affected accordingly and are reviewed on an individual basis. We decided to classify advanced dental caries according to the root canal treatment. The analysis method using DMFT index, which was mainly used in previous studies, is a cross-sectional assessment of accumulated information about the oral health of patients. While this procedure has methodological convenience, it may not be an appropriate way to assess the risk factor for ongoing active diseases such as tooth decay. Therefore, we tried to devise an alternative method using the patient's symptom manifestation and subsequent visits to the dentist to compare factors affecting the incidence of advanced dental caries, especially the association with the patient’s BMI as a factor determining incidence of dental caries in general. Dental caries, including pulpal and periapical disease was coded as K02, and K04 on the International Classification of Diseases, Tenth Revision, Clinical Modification (ICD-10-CM) [19]. If these diagnostic codes were recorded more than three times a year, we defined it as a patient with advanced dental caries.

The purpose of this study was to investigate the significance and association between incidence of overweight, obesity and advanced dental caries requiring root canal treatment in Korean adults using diagnostic code and frequencies of visiting dental health professional. We also tried to evaluate the effect of lifecycle behavior such as drinking, smoking, and exercise habits on this association and data review.

## Materials and methods

### Survey overview and study subjects

**Fig 1:**
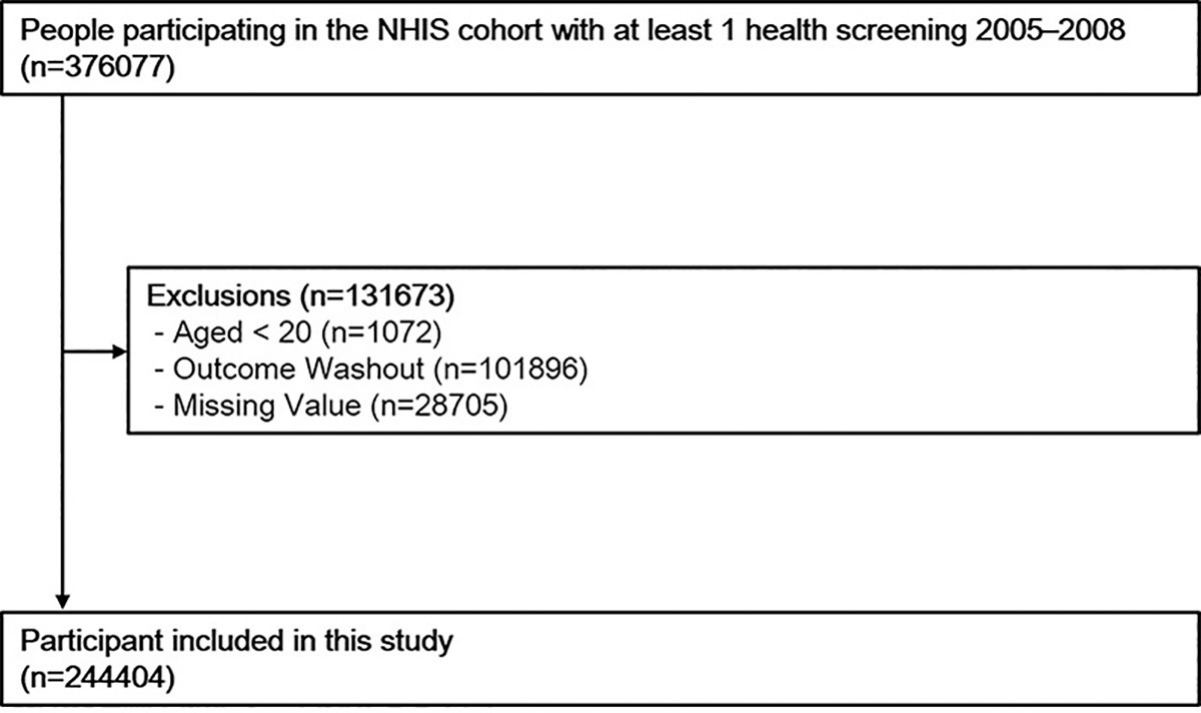
A flow chart showing the selection of study participants.

This study used data from the Korean National Health Insurance Service (KNHIS), a government-affiliated agency under the Korean Ministry of Health and Welfare that manages and supervises all medical activities and procedures for healthcare in Korea. The KNHIS contains notations on the eligibility, medical treatment, health examination, and medical care institution databases of all patients within that database who received care for the years reviewed. The original collection of data and the secondary analysis of data was also approved by the Institutional Review Board of the Seoul St. Mary’s Hospital, Catholic University of Korea (KC16EISI0332). Our research is based on the use and analysis of anonymized data owned by the KNHIS at a certain cost. Generally, the anonymized and de-identified information was used for analyses, and therefore informed consent was not required in that case. The original data is subject to deletion after the research is finished. The permits for this protocol and procedure are given in NHIS-2016-2-079. Using KNHIS data, we customized a sample size of 1,025,340 subjects selected by a systematic sampling method to generate a high level representative sample from the total Korean population existing in 2005 [20]. The systematic sampling method was applied to the number of proportioned samples within 1,476 floors according to the age-gender-qualification-income tier level combination. In other words, the populations in the strata were sorted based on the annual total medical expenses, and then the stratification was performed. As a final sample, the systematic extraction process was repeated in each of 1,476 floors, and the final selection of a sample representative of the distribution of total medical expenses in the floors was used. Of the 376,077 participants who underwent a health examination at least one time from 2005–2008, those aged <20 years (n = 1072) were excluded from the study. There were 101,896 outcome washouts from 2002 to the day of the examination and 28,705 missing values which were also excluded. Therefore, the final number of people included in the study was noted at 244,404. The dataset also includes information on all medical claims filed and check-ups attended by participants from 2005 to 2014. The process for selecting participants is shown in Fig 1. The participants' age, gender, smoking status, alcohol consumption, physical exercise, diabetes, hypertension, dyslipidemia, height and weight were collected and organized.

### Definition of advanced dental caries

In Korea, the National Health Insurance is mandated by law as applicable for all citizens since 1977, and the medical statement, medical history, and prescription details are established as a KNHIS database. This vast sample cohort database is provided to researchers to support academic research, to help establish national policies in health care, and ultimately to be a cornerstone for improving public health and the general advancement of patient welfare in this country. Most people included in KNHIS, pay a certain amount of income every month. In this case, some of the medical expenses are subsidized by the KNHIS. Therefore, all of the data is charged and recorded to the KNHIS when the medical expenses are noted to have occurred. In the dental field, these subsidies include extraction, periodontal treatment, root canal treatment, amalgam restoration, and glass ionomer restoration, and do not include restoration including composite resin, inlay, onlay, and the installation of a crown. Also, in the case of root canal treatment, there are criteria and conditions in which subsidy is supported for each treatment action, whereby root canal treatment is usually completed through 2 or 3 visits. Therefore, we can screen patients who have undergone root canal by using the methods we have used as noted above.

We defined advanced dental caries requiring root canal treatment as a diagnostic code of K02 or K04 more than three times a year. In this case, the diagnostic code K02 includes all caries. If a patient visits more than three times a year, it was noted that the patient might have advanced dental caries or rampant caries. Our data is derived from KNHIS, which is based on medical bills paid through national health insurance. In Korea, dental treatments such as composite, inlay, and crown restorations are not supported by the National Health Insurance. Therefore, these treatments are not recorded in KNHIS's database. This means that, most cases recorded more than three times as K02 or K04 can be expected to be root canal treatment. In the case of code K04, it is seen as a diagnosis ranging from reversible pulpitis to apical cyst including pulp necrosis, apical periodontitis, and periapical abscess. Notably, three or more visits are usually considered to conduct root canal treatment. Most causes of these pulpal diseases requiring root canal treatment are seen to be due to advanced dental caries or trauma. Dental trauma refers to structural damage of a tooth. It is further classified as fracture, concussion, or dislocation of tooth or root mainly diagnosed as s025 or s032. Thus, it can be excluded in this study. The data were retrieved on sex, birthdate, and diagnostic codes. Additionally, it was noted that if these codes were billed more than 3 times a year, we defined this person as having advanced dental caries.

### Health and lifestyle behaviors

In additional to age and sex, the health examination revealed information on advanced dental caries and BMI. The BMI was calculated according to the formula: weight (kg) / height^2^ (m^2^). The BMI was also categorized into underweight, normal (BMI < 23) and overweight (BMI ≥ 23) as was noted according to the categorization and recommendation of the Korean Society for the Study of Obesity [21]. As described in detail previously [6], the smoking status was categorized into three groups: non-smokers, current smokers who had smoked 100 cigarettes or more in their lifetime, and ex-smokers who had smoked in the past, but who had since quit at least one month. The measure of alcohol consumption status was categorized into two groups: non-drinker, and those who drank more than two-to-three times a month. Finally, the exercise status was also divided into two groups: non-exerciser, physical activity performed for at least 30 minutes at more than four times a week.

### Statistical analysis

The SAS software package version 9.3 (SAS Institute, Cary, NC, USA) was used for statistical analysis in this study. Additionally, a multivariate adjusted Cox regression analysis was conducted to examine the hazard ratio (HR) and 95% confidence interval (CI) for the association between advanced dental caries and BMI. To this end, the calculations were made adjusting for age, smoking status, alcohol consumption, and exercise. For each Cox regression analysis, a p-value for linear trend across categories was calculated by introducing the ordinal variable in the model. In this case, a p-value < 0.05 was considered statistically significant.

## Results

The general characteristics of the study population and subgroups are summarized in Table 1. Between the beginning of 2005 and the end of 2014, there were 81,971 incident cases of advanced dental caries as noted in the data. The incidence density 60.9 cases per 1,000 person-years. Among a total of 244,404 participants, 111,655 (35.74%) had normal weight while 132,749 (35.14%) were shown to be overweight (including those already marked with obesity). The average age of participants was 45.44, and the ratio of male to female was 1: 1.06 (118,639 male and 125,765 female).

**Table 1:** Comparison of clinical characteristics according to BMI.

BMI category (kg/m^2^)	< 23	≥ 23
No of individuals	111655	132749
**Age (≥ 65)**	12198 (10.92)	15819 (11.92)
**Sex (Male)**	47954 (42.95)	77811 (58.62)
**Smoking**		
Non	81155 (72.68)	88673 (66.80)
Ex.	3811 (3.41)	7509 (5.66)
Current	26689 (23.90)	36567 (27.55)
**Alcohol consumption**	50766 (45.47)	64359 (48.48)
**Exercise**	44146 (39.54)	64152 (48.33)
**Current Diabetes**	5231 (4.68)	12271 (9.24)
**Current Hypertension**	15981 (14.31)	40162 (30.25)
**Current Dyslipidemia**	7149 (6.40)	17818 (13.42)
**Height**	162.75 ± 8.84	163.66 ± 9.63
**Weight**	55.2 ± 7.48	69.44 ± 10.26

Data are presented as the means ± SE, or % (SE). BMI: body mass index, SE: standard error.

Table 2 shows the results of the association between the incidence of advanced dental caries and BMI parameters by regression analyses after adjusting for the applicable covariates. Being over 65 years old (which represented the elderly; in Korea, the working age population has been set as 15–64 years old since 1964, and this notation has remained to the present) and female were significant risk factors in both age-adjusted and multivariable-adjusted models. For non-smoking participants, the increase in HR of advanced dental caries due to changes in BMI shown to be decidedly was more pronounced.

**Table 2:** Hazard ratios for advanced dental caries in multivariable Cox regression analysis.

		BMI category	N	event	Total follow-up	Incidence rate^1^	Hazard ratio (95% CI)	
							Crude	Multivariables adjusted^2^
**TOTAL**		< 23	111655	35059	620197.50	56.53	1 (reference)	1 (reference)
		≥ 23	132749	46912	725022.85	64.70	1.14 (1.13, 1.16)	1.10 (1.09, 1.12)
**Age**	< 65	< 23	99457	32184	557054.42	57.78	1 (reference)	1 (reference)
		≥ 23	116930	41819	643606.98	64.98	1.12 (1.11, 1.14)	1.04 (1.02, 1.05)
	≥ 65	< 23	12198	2875	63143.08	45.53	1 (reference)	1 (reference)
		≥ 23	15819	5093	81415.87	62.56	1.37 (1.31, 1.44)	1.25 (1.20, 1.31)
**Sex**	Male	< 23	47954	15534	266458.27	58.30	1 (reference)	1 (reference)
		≥ 23	77811	26651	434192.59	61.38	1.05 (1.03, 1.07)	1.05 (1.03, 1.07)
	Female	< 23	63701	19525	353739.23	55.20	1 (reference)	1 (reference)
		≥ 23	54938	20261	290830.26	69.67	1.26 (1.23, 1.28)	1.19 (1.17, 1.22)
**Smoking**	Non	< 23	81155	25114	451908.80	55.57	1 (reference)	1 (reference)
		≥ 23	88673	31886	479726.54	66.47	1.19 (1.17, 1.21)	1.03 (1.00, 1.05)
	Ex	< 23	3811	1210	21216.39	57.03	1 (reference)	1 (reference)
		≥ 23	7509	2593	41126.39	63.05	1.10 (1.03, 1.18)	1.14 (1.12, 1.16)
	Current	< 23	26689	8735	147072.30	59.39	1 (reference)	1 (reference)
		≥ 23	36567	12433	204169.92	60.90	1.03 (1.00, 1.06)	1.09 (1.02, 1.17)
**Drinking**	No	< 23	60889	19237	335971.80	57.26	1 (reference)	1 (reference)
		≥ 23	68390	25082	365925.02	68.54	1.20 (1.17, 1.22)	1.16 (1.14, 1.19)
	Yes	< 23	50766	15822	284225.70	55.67	1 (reference)	1 (reference)
		≥ 23	64359	21830	359097.83	60.79	1.16 (1.14, 1.18)	1.17 (1.15, 1.19)
**Exercise**	No	< 23	67509	20454	374945.67	54.55	1 (reference)	1 (reference)
		≥ 23	68597	23838	373728.74	63.78	1.17 (1.15, 1.18)	1.14 (1.12, 1.16)
	Yes	< 23	44146	14605	245251.82	59.55	1 (reference)	1 (reference)
		≥ 23	64152	23074	351294.11	65.68	1.14 (1.12, 1.16)	1.15 (1.13, 1.17)

Data are presented as HR (95% confidence interval).

^1^Incidence rates are expressed as number per 100 person-years

^2^Adjusted for age, sex, smoking, drinking and exercise

BMI: body mass index, HR: Hazard ratio, CI: confidence interval

**Fig 2:**
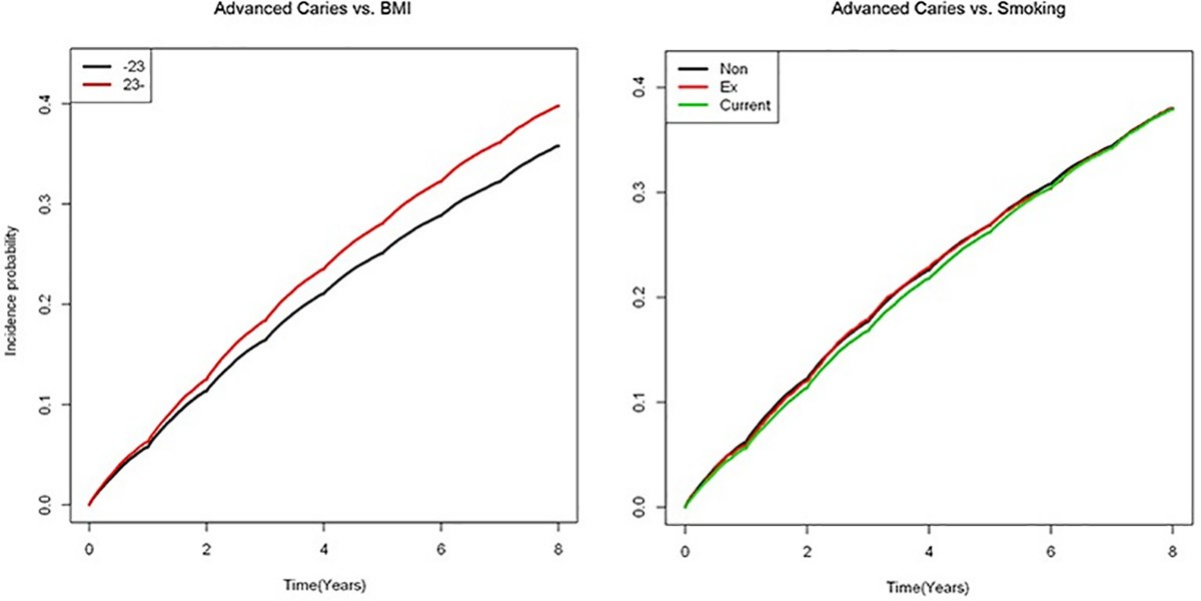
Cumulative hazards (incidence probability) of advanced dental caries.

In the selection of variables, we selected the available variables in KNHIS data that are expected to influence the change in BMI. In addition, lifecycle behaviors including smoking status, alcohol consumption status, and exercise were included in the study, and the analysis showed that smoking was considered to be a statistically significant factor in this case. The HR for advanced dental caries was stratified by BMI level and by the smoking status in age- and multivariable- adjusted models (Table 2, Fig 2). It is noted that in overweight people, the HR of advanced dental caries became more vulnerable. In the normal weight group, it was also shown that categories of smoking impacted the HR (p<0.05). The risk for advanced dental caries in normal and underweight groups with smoking was significantly increasing compared to the group with the same BMI without smoking (Table 3).

**Table 3:** Hazard ratios for advanced dental caries to body mass index in multivariable Cox regression analysis.

BMI	Smoke	N	Event	Total follow-up	Incidence rate^1^	Hazard ratio^2^ (95% CI)
23 <	Non	81155	25114	451908.8	55.57	1^3^ (ref.)
	Ex	3811	1210	21216.39	57.03	1.05 (0.99,1.11)
	Current	26689	8735	147072.31	59.39	1.12 (1.09,1.15)
≥ 23	Non	88673	31886	479726.53	66.47	1.14 (1.12,1.16)
	Ex	7509	2593	41126.39	63.05	1.15 (1.10,1.20)
	Current	36567	12433	204169.92	60.9	1.16 (1.13,1.19)

Data are presented as HR (95% confidence interval).

^1^Incidence rates are expressed as number per 100 person-years

^2^Adjusted for age, sex, smoking, drinking and exercise

BMI: body mass index, HR: Hazard ratio, CI: confidence interval

^3^Reference group

## Discussion

This large population-based national cohort study showed that overweight and obese people were more likely to develop advanced dental caries, such as requiring root canal treatment independent of confounding variables in Korean adults. The positive association between overweight, obesity and advanced dental caries were estimated as being more prominent in elderly people over 65 years of age and in women. Among the health and lifecycle behaviors, smoking or not was found to be one of the factors affecting the results.

The present observational analysis is meaningful in that it is the first study surveying large number of adults consistent with previous reports, which were performed mainly in children and adolescents [3,22–24]. Furthermore, Korea’s NHIS database with a highly representative cohort of the most Korean people, contains a large sample size and detailed information which is useful in this and similar reports, such as the physical characteristics and lifecycle behaviors including drinking, smoking status, and exercise [20]. We also used an alternative method to screen advanced dental caries requiring root canal treatment, which is clinically important to distinguish among clinicians and also with patients.

We used the diagnostic code according to ICD-10 and visit frequency to define advanced dental caries in this study. Previous studies on dental caries and obesity have mainly used the number of teeth and DMFT index and are known to have some limitations [12]. When using the number of remaining teeth, the cause of extraction may be not only dental caries but also periodontitis, tooth fracture, trauma, other bone diseases, or for prosthetic reasons. In this sense, the total caries experience or DMFT index is defined as a sum of decayed, missing, and filled teeth (WHO, 2013). This index includes all previous or present permanent decayed teeth which illustrate a cumulative caries experience. This means that the participant had experience with past caries. However, it has a blind spot to determine the incidence of active disease like advanced dental caries. In particular, we conducted an observational study on BMI and specific diseases using data from the National Health Insurance Corporation, and also found that the definition using the diagnostic code and number of visits was significantly more useful.

The other distinctive feature of this study was the first description regarding such a positive association in the elderly. With increasing age, in most patient populations the number of teeth experiencing caries accumulates while the number of remaining teeth decreases due to various causes. The effect of caries on the number of teeth and DMFT index is gradually diluted. Therefore, an alternative method is needed to evaluate caries to provide strategies to increase helping patients keep their teeth during older ages. Our approach using diagnostics and frequency of visits can show possible relationship. Unlike changes in eating habits in young people due to changes in the social-economic environment, changes in eating habits in the elderly may be related to the reduction of muscles caused by aging, weakening of masticatory and the number of the individual’s residual teeth. This leads to changes in dietary habits that rely on high nutrition, high calorie and soft foods, and these foods are more cariogenic due to their high viscosity and long oral residence time. In a previous study about oral health of the elderly, it was found that the lower the number of teeth, the lower the BMI [25]. Our study, which excluded the effect of the number of teeth, showed a higher association between BMI and progressive dental caries in most cases.

The greater influence in women may be due to early eruption and hormonal fluctuations as has been reported in previous studies [26,27]. Early eruption results in prolonged exposure to cariogenic oral environment while the incidence of hormonal fluctuations can affect saliva composition and flow rate. In addition, clinical studies have shown that the female hormone (estrogen) level is elevated during immune response and that women can be protected against pathogens in life-history events such as puberty, menstruation and pregnancy [26]. These hormonal fluctuations in women have a greater impact on the development of advanced cavities and lead to a higher association with BMI changes, as has been shown in the study results.

Smoking was also associated with an increase in advanced dental caries in the patients in our study. Although, the direct association between smoking and an increased incidence of dental caries remains controversial [28]. However, smoking is known to decrease pH and effect the buffering power of saliva, which then acts as an agent that works shifting bacterial population from bacilli to streptococci [29–31]. Some studies have further indicated that smoking is one of contributing factors of dental caries in adults [32] as well as elder people and teenagers [33–35]. In this respect, our results are in line with these studies. However, in the study group with a BMI of 23 or higher, the smoking effect was considered to be relatively less prominent. It is concluded that this result is most likely because of the influence that from increasing BMI has a greater impact on advanced dental caries than which of smoking.

However, this study has some noted limitations. First of all, the dietary habits and oral hygiene were not considered because only the evaluation of BMI and advanced dental caries were compared. The KNHIS database, based on our research, did not include information about dietary habits or oral hygiene, and for this reason we could not consider it. Second, there may be some blind spots in the new method using diagnostic code and frequencies of visit. For example, pulpal disease caused by cracks can be included as variable reasons for the root canal treatment, but were also not considered for this study. And patients who developed systemic disease might have more frequent contacts with the dental care system which might induce surveillance bias, and this was also not considered as a factor in this study. Our study has a limitation with BMI categorization. As in previous studies that reported the relationship between BMI and dental caries, the distinction between underweight and normal weight groups may be necessary to clarify the results [36]. While the purpose of this study is to devise a new method, it should be considered in future studies.

In other previous studies [5,37–39], it has been shown that the trend of the intake of sugar from beverages and polysaccharides in children and adolescents is increased over the years, while the intake of vegetables and proteins is decreased while obesity and dental caries are increased simultaneously. These findings were also assumed to be relevant and seen in Korean adults. This conclusive result can be explained by the increase in the ratio of sugar intake to total energy intake in the National Health and Nutrition Survey [40]. In addition, when the frequency of food intake is increased and the intake interval is decreased, oral pH is kept low which provides an environment which is shown to be favorable for the growth and spread of *Streptococcus mutans*, a causative type of bacteria for dental caries. In other words, frequent food intake plays a major role in the development or progression of dental caries, as well as in the progression of the development of obesity in all population demographics. Increases in obesity and dental caries due to increased consumption and frequency of drinking beverages and refined sugars have been found in many studies [40–42]. However, unlike studies in which adolescent physical activity positively affects oral health [43], our study showed no association between exercise habits and an individual’s BMI (*p = 0*.*0045*).

Our findings show that increased BMI affects the development of advanced dental caries in the elderly population above 65 years, especially women. Unlike previous studies using DMFT, this means that caries that require root canal treatment are more likely to occur, excluding missing teeth from past experiences. Due to the extension of life expectancy, it is essential in modern societies to consider growing elderly populations and their health problems, not only in Korea but also globally. Obesity is one of those concerns, so many studies are ongoing, and our research is in this context. Clinicians treating older patients should consider not only oral diseases, but also various systemic diseases. As our findings show that BMI and advanced dental caries increase in older women, associations with various causative factors and diseases are being published. If dentists learn these associations and care about them when they see a patient, they will be able to provide the patients with better care along with the treatment of oral disease.

This study suggests an alternative method for evaluating dental caries as found in a review of cases seen in epidemiologic studies. This method can also be used to study the relationship between advanced dental caries and other medical conditions or treatments, which were included in the KNHIS database. The diagnosis according to ICD-10 and appropriate methodological modifications are thought to be applicable not only in Korea, but will be used also in other countries with large cohort data. For example, if the method is used in the National Wide Inpatient Sample (NIS) in the United States and the National Health Insurance Research Database (NHIRD) in Taiwan, there is a possibility that research in a wider variety of relevant and additional fields could be conducted. In addition, there is another large-scale cohort database, the National Health and Nutrition Survey, which contains information on dietary habits and oral hygiene that our study for lack of time did not review. In the future, if these two cohort data are combined, the research will expand our horizons of various dental diseases in the oral cavity, and their effect on a patient’s overall health, as well as the effect of dental caries in other health areas affecting a patient’s physiology.

## Conclusions

This study is a nationwide cohort study to clarify the relationship between advanced dental caries requiring root canal treatment and the incidence of associated patient status being overweight and obesity. Using a high representative KNHIS database and alternative method, it is noted that high BMI causes the incidence of advanced dental caries, which is more pronounced in women and older than 65 years. Considering the various lifecycle behaviors, smoking was seen to be a substantial factor which influenced the development of advanced dental caries.
